# Nuclear Medicine Imaging in Pediatric Neurology

**DOI:** 10.4274/mirt.49389

**Published:** 2016-02-10

**Authors:** Ümit Özgür Akdemir, Lütfiye Özlem Atay Kapucu

**Affiliations:** 1 Gazi University Faculty of Medicine, Department of Nuclear Medicine, Ankara, Turkey

**Keywords:** Single-photon emission computed tomography, Positron emission tomography, Neurology, Epilepsy, brain neoplasms, Tc-99m HMPAO, F-18 FDG

## Abstract

Nuclear medicine imaging can provide important complementary information in the management of pediatric patients with neurological diseases. Pre-surgical localization of the epileptogenic focus in medically refractory epilepsy patients is the most common indication for nuclear medicine imaging in pediatric neurology. In patients with temporal lobe epilepsy, nuclear medicine imaging is particularly useful when magnetic resonance imaging findings are normal or its findings are discordant with electroencephalogram findings. In pediatric patients with brain tumors, nuclear medicine imaging can be clinically helpful in the diagnosis, directing biopsy, planning therapy, differentiating tumor recurrence from post-treatment sequelae, and assessment of response to therapy. Among other neurological diseases in which nuclear medicine has proved to be useful are patients with head trauma, inflammatory-infectious diseases and hypoxic-ischemic encephalopathy.

## Abstract (tr)

Nükleer tıp görüntüleme nörolojik hastalığı olan çocukların klinik yönetimine sağladığı önemli bilgiler ile katkı yapmaktadır. Medikal tedaviye dirençli epilepsi hastalarında cerrahi tedavi öncesinde epileptojenik odağın belirlenmesi pediyatrik nörolojide nükleer tıp görüntülemenin en sık endikasyonudur. Temporal lob epilepsisinde nükleer tıp görüntüleme özellikle manyetik rezonans görüntüleme bulguları normal olduğunda veya bulgular elektroensefalografi bulguları ile uyumsuz olduğunda yararlıdır. Beyin tümörü tanılı pediyatrik hastalarda nükleer tıp görüntüleme tanıda, biyopsi yerinin belirlenmesinde, tedavinin planlanmasında, rezidü/rekürren tümör ile tedaviye bağlı değişikliklerin ayırıcı tanısında ve tedaviye yanıtın değerlendirilmesinde kullanılır. Nükleer tıp görüntülemenin yararlı olduğu gösterilen diğer nörolojik hastalıklar arasında kafa travması, enflamatuvar-enfeksiyöz hastalıklar ve hipoksik-iskemik ensefalopati yer alır.

## INTRODUCTION

Nuclear medicine offers noninvasive imaging methods, namely single-photon emission computed tomography (SPECT) and positron emission tomography (PET) to study functional changes associated with neurological disorders (1). The most common indication for nuclear medicine imaging in the pediatric population is epilepsy. That is why; nuclear medicine imaging in epilepsy is covered in more detail in this review article, while other indications are briefly mentioned. Additionally, a general description of nuclear medicine imaging methods will be provided.

## METHODOLOGICAL ISSUES

**Brain Single-Photon Emission Computed Tomography**

SPECT is the specific technique used to acquire the three-dimensional distribution of a radiopharmaceutical using a gamma camera. Regional cerebral perfusion can be measured by using technetium-99m (Tc-99m) labeled compounds such as ethyl cysteine dimer (ECD) and hexamethyl propylene amine oxime (HMPAO). These agents cross the blood-brain barrier due to their lipophilic character, and are retained within the brain by their conversion into hydrophilic compounds proportional to regional cerebral blood flow. Their extraction from plasma to brain occurs rapidly within 1-2 minutes and this initial tracer uptake remains almost unchanged for several hours that allow acquisition of a constant image of regional cerebral blood flow at the time of tracer injection. However, there are several differences between these two radiopharmaceuticals in respect to in vitro stability, uptake mechanism, cerebral distribution and dosimetry. When epilepsy is considered, the most important of these factors is the in vitro stability that is 30 min after reconstitution for unstabilized Tc-99m-HMPAO, 4 h for stabilized Tc-99m-HMPAO, and 6 h for Tc-99m-ECD (2,3). The chemical stability of Tc-99m-ECD has led to an increased use of this agent in ictal brain SPECT imaging in epilepsy (3,4). In addition to cerebral perfusion tracers, other radiopharmaceuticals can also be used for SPECT imaging of neurotransmission and amino acid metabolism. The most commonly used of such agents are iodine (I)-123-labelled ß-CIT and FP-CIT that bind with high affinity to the dopamine transporter (5) located in the membrane of the presynaptic nigrostriatal nerve terminals, I-123-labelled IBZM and epidepride for imaging D2-like dopaminergic receptors, and I-123-labelled Iodo-α-methyl-L-tyrosine (IMT) for imaging brain tumors (6,7).

## BRAIN POSITRON EMISSION TOMOGRAPHY

Glucose metabolism provides the major energy supply required for brain function, therefore is highly correlated with neuronal activity. Pathophysiologic changes in neuronal activity in disease states cause changes in glucose metabolism that can be detected via the radiolabelled glucose analogue fluorine-18 fluorodeoxyglucose (F-18 FDG), which is currently the most commonly used tracer for brain PET imaging. The most common indications for brain F-18 FDG PET imaging include preoperative evaluation of partial epilepsy patients to identify the functional deficit zone, early diagnosis and differential diagnosis of dementing disorders, differential diagnosis of cerebral tumors, and detection of viable tumor tissue following therapy (8). There are numerous PET radiotracers for other brain functions due to their better radiolabeling properties such as carbon-11, nitrogen-13, and oxygen-15 (9). However, their use in childhood neurological disorders are limited and beyond the scope of this review article.

## PATIENT PREPARATION AND ACQUISITION

Since brain perfusion and metabolism are coupled with neuronal function, radiotracer injection should be done in a controlled environment to keep neuronal activation at a minimum. Patients should be positioned comfortably in a quiet, dimly lit room, with an intravenous line inserted at least 10 min before administration of the radiotracer. Patients should be instructed not to speak, read or be active otherwise at the time of injection and during the uptake phase, which is at least 20 min for F-18 FDG (8). Medications known to affect brain perfusion and metabolism should be withheld before imaging, if there is no clinical contraindication. In the evaluation of epilepsy, continuous video-electroencephalogram (EEG) monitoring starting (ideally) 2 h before injection and continuing during the uptake phase is recommended in order to relate the injection time exactly to the time point of behavioral and electrical seizure onset for accurate image interpretation. For the ictal perfusion SPECT study, the tracer should be injected as soon as possible after the onset of seizure. Therefore, the radiopharmaceutical should be prepared and stored in the epilepsy monitoring unit to ensure the quickest possible injection time. The patient should be supervised continuously during the scanning procedure. If sedation is required in uncooperative patients, it should be given after completion of the radiotracer uptake, preferably only a few minutes before starting data acquisition.

## INTERPRETATION OF IMAGES

**Figure 1:**
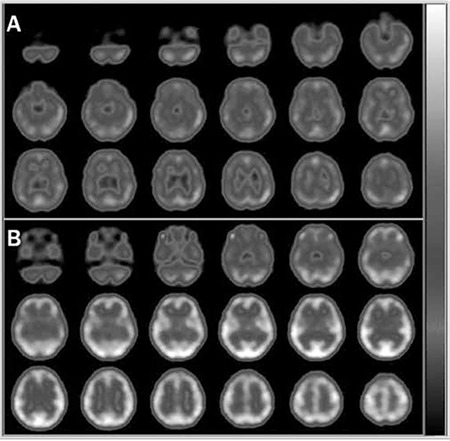
Interictal (A) and ictal (B) Tc-99m hexamethyl propylene amine oxime brain perfusion single-photon emission computed tomography (SPECT) studies of a 7-year-old female with absence seizures. Electroencephalogram showed bilateral synchronous symmetrical epileptic activity and the magnetic resonance imaging was normal. Ictal SPECT showed global increase in cerebral perfusion relative to the interictal examination. Counts in each scan were normalized to their cerebellum that were set equal between the two scans so that the global increase in cerebral perfusion could be detected

**Figure 2:**
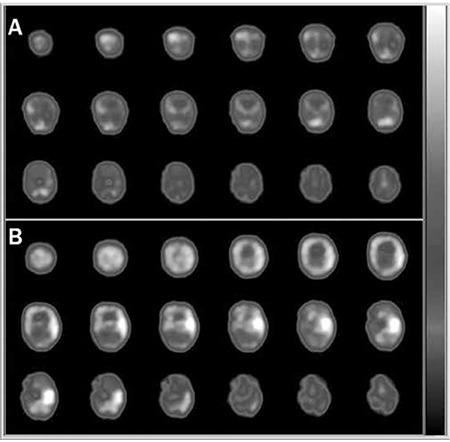
Interictal (A) and ictal (B) Tc-99m hexamethyl propylene amine oxime brain perfusion single-photon emission computed tomography (SPECT) studies of a 12-d-old infant with intractable seizures. The interictal SPECT showed an area of hypoperfusion in the left temporal cortex, whereas the ictal SPECT revealed prominent hyperperfusion in the same area. Additionally, in the interictal brain perfusion SPECT, higher tracer uptake in the primary sensorimotor cortex, thalamus, and cerebellum was observed that is typical for the neonatal period (24,25)

The interpretation of brain perfusion and metabolism data essentially depends on visual assessment of images. When comparing two different brain datasets of a patient, such as ictal and interictal studies, it is important to normalize the counts in each dataset using a reference region (Figure 1). Although it is not accepted as the standard for all conditions, the cerebellum is generally used as a reference for this normalization (2). Additional quantitative analytic methods are commonly used and have been shown to improve results of visual assessment (10,11,12,13). Image subtraction is usually applied for the comparative analysis of two brain imaging datasets of the same patient acquired in two different conditions. In this analysis, a baseline study is subtracted from an activation study after spatial and count normalization of the images. Subtraction ictal SPECT co-registered to magnetic resonance imaging (MRI) called SISCOM is the most frequently used subtraction analysis technique that is considered as the gold standard for quantitative assessment in ictal/interictal analysis of epilepsy patients. It enhances the sensitivity and spatial accuracy of ictal SPECT and enables correlation of findings of functional imaging to structural imaging (14,15,16,17,18,19,20). Other quantitative methods include 3-D surface projection algorithms, which are useful in detection of cortical disease and voxel-based statistical parametric mapping (SPM) (12,21,22,23). Using an institutional control group, SPM analysis could be used to both lateralize and localize the epileptogenic seizure foci on interictal F-18 FDG PET studies and increase confidence levels for the interpretation of images by the observers with different experience levels. In general, these methods allow objective analysis of individual patient data as compared to a normal database. Global and regional brain perfusion and metabolism display an age-dependent variation parallel to the ongoing brain maturation during childhood that persists until 16-18 years of age (24,25). Regionally, brain perfusion and glucose metabolism is highest in primary sensory and motor cortex, cingulate cortex, thalamus, brain stem, cerebellar vermis, and hippocampal region in the newborn (Figure 2A, 2B). Glucose metabolism increases in the parietal, temporal, and primary visual cortex, basal ganglia and cerebellar hemispheres by 2 to 3 months of age, and in the frontal cortex by 6 and 12 months of age. Glucose metabolism becomes similar to that of young adults by 1 year of age. It is necessary to take these age-dependent changes in brain perfusion and metabolism into consideration during the evaluation process.

## NUCLEAR MEDICINE IMAGING IN PEDIATRIC EPILEPSY

Epilepsy is the most common indication for nuclear medicine studies among pediatric neurological diseases. Approximately 20-30% of patients with epilepsy have intractable or refractory disease that can be treated with surgery (26,27). A patient’s eligibility for surgery is decided on after a comprehensive assessment that includes clinical history and examination, MRI, EEG, SPECT and PET studies, and if necessary, invasive EEG monitoring. The major aim of this pre-surgical assessment is localization of the epileptogenic focus, since the success of surgery depends on the accurate recognition and resection of this region. Epilepsy is characterized by the presence and propagation of abnormal neural activity. The rationale for nuclear medicine imaging in epilepsy is to visualize this abnormal activity. As the uptake and trapping of regional cerebral blood flow tracers within the brain is rapid, the SPECT image represents the regional cerebral blood flow during the seizure, although the image is acquired after the seizure. The epileptogenic focus during a seizure shows a focal area of significantly increased perfusion. The critical issue about an ictal SPECT scan is the timing of tracer injection. If the injection can be done promptly at the beginning of a seizure, the probability of visualizing an epileptogenic focus would increase. If the injection is delayed, the probability of localizing the epileptogenic focus would decrease due to seizure propagation (27). Since cerebral perfusion and metabolism are coupled during a seizure, ictally, both the regional cerebral perfusion and metabolism are increased. However, brain F-18 FDG PET is not considered as a suitable method for ictal imaging due to its longer uptake time and poor temporal resolution. Due to the rapidness of tracer uptake, brain perfusion SPECT is the preferred method for ictal imaging. A successful tracer injection early in the beginning of a seizure gives the best result with respect to localization of the ictal onset zone. A delayed injection during the seizure may show areas with increased tracer uptake related to the spread of electrical discharge from seizure propagation and may fail to localize the epileptogenic focus. Interictal studies are based on the observation that regional cerebral perfusion and metabolism are reduced at the epileptogenic focus during the interictal period. However, the findings of an interictal F-18 FDG PET scan may be confusing if the patient has a seizure close to the time of tracer injection. Therefore, EEG monitoring is recommended to trace possible clinical or subclinical seizures that may occur during the F-18 FDG uptake period. According to semiology, seizures are classified into generalized and partial seizures. Partial seizures are also separated into complex partial and simple partial seizures according to whether they are associated with loss of consciousness and awareness or not. In patients with primary generalized epilepsy, such as absence and pure photosensitive epilepsy syndromes, a global increase in brain perfusion during the ictal study is generally observed (28,29). The interictal studies in these patients showed frontal hypoperfusion that suggests an altered frontal lobe function in patients with childhood absence and pure photosensitive epilepsy syndromes. Although functional imaging findings may provide the understanding of pathophysiologic changes in primary generalized epilepsy, patients with complex partial seizures are the ones who would benefit most from functional imaging.

**Figure 3:**
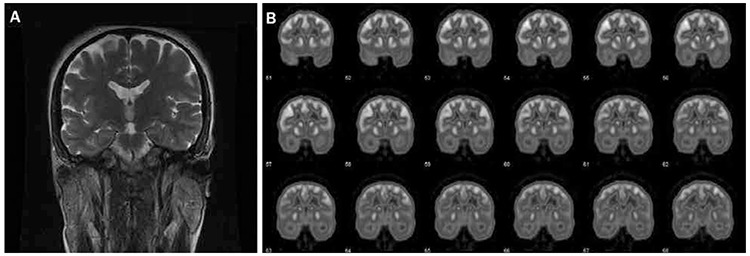
Coronal magnetic resonance imaging (MRI) view (A) and interictal fluorine-18 fluorodeoxyglucose (F-18 FDG) positron emission tomography (PET) study (B) of a 27 year old male patient with intractable complex partial seizures. The electroencephalogram recordings of the patient displayed bilateral temporal lobe abnormalities, while the MRI was normal (nonlesional temporal lobe epilepsy). Interictal F-18 FDG PET study showed hypometabolism on the right temporal lobe. At the postoperative (right anterior temporal lobectomy) follow up, the patient was seizure free (Engel I outcome)

Epilepsy patients with normal MRI findings (non-lesional epilepsy) can benefit from nuclear medicine imaging (20,30,31,32,33). In our institutional database of 141 temporal lobe epilepsy (TLE) patients who underwent surgery, PET was able to lateralize the epileptogenic zone in all of the non-lesional TLE patients (n=24) (Figure 3) (34). At follow-up after surgery, seizure control (Engel I) was achieved in 19 of these 24 patients and the outcome was similar to patients with mesial temporal sclerosis on MRI. Nuclear medicine can also help in lateralizing or localizing the epileptogenic focus when the findings of MRI are discordant with EEG and seizure semiology (31). In that case, if a possible epileptogenic focus was detected on SPECT and/or PET, further evaluation with invasive EEG monitoring would be necessary for surgical planning. The abnormality detected on SPECT and/or F-18 FDG PET can be used as a guide for placement of electrodes for invasive EEG monitoring. Nuclear medicine imaging can also help to localize the epileptogenic focus in case of multiple structural abnormalities on MRI by indicating which one of the lesions may be the epileptogenic focus (35). In the presence of concordant findings on clinical evaluation, EEG recordings and MRI, nuclear medicine imaging is not indicated.

## TEMPORAL LOBE EPILEPSY

**Figure 4:**
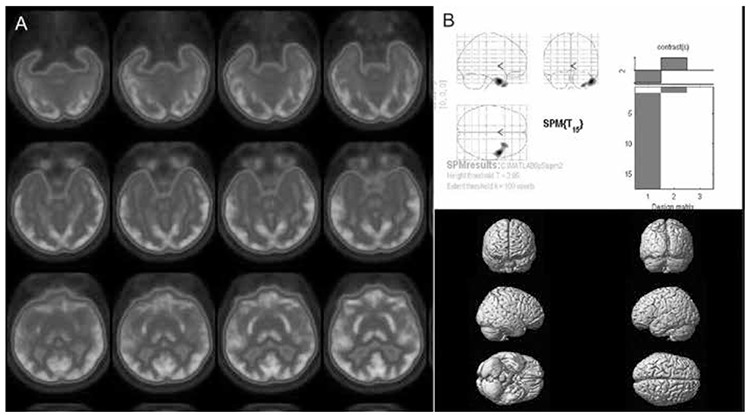
Interictal fluorine-18 fluorodeoxyglucose positron emission tomography study of a temporal lobe epilepsy patient with complex partial and secondary generalized tonic-clonic seizures. The axial views (A) show right mesial and lateral neocortical hypometabolism visually that was also evident on the quantitative analysis by statistical parametric mapping (B) that showed significantly reduced metabolism on the right temporal lobeSPM: Statistical parametric mapping

In pediatric TLE, the most common underlying pathologies are mesial temporal sclerosis, focal cortical dysplasia and developmental tumors (36). The usual interictal F-18 FDG PET finding in TLE is ipsilateral temporal hypometabolism that is observed in 85% of cases (Figure 4) (26,33). In lesional epilepsy, the extent of hypometabolism is generally greater than the structural lesion (37). Additional regions with milder hypometabolism may also be observed in the entire ipsilateral temporal lobe, extratemporal regions such as ipsilateral frontal and parietal lobes, thalamus, basal ganglia, and the contralateral temporal lobe. This wide area of hypometabolism is believed to represent the epileptogenic network involved in seizure spread. Glucose metabolism in these remote areas shows normalization following surgery that may be due to the release of metabolic inhibition by the intercortical pathways (27,38). Patients with bilateral temporal lobe hypometabolism have a worse prognosis for seizure remission after surgery in comparison to patients with unilateral temporal lobe hypometabolism (30,39). Additionally, in patients with unilateral temporal lobe hypometabolism, the probability of becoming seizure-free was higher for patients with mesial temporal hypometabolism than patients with neocortical temporal lobe hypometabolism (40). The lateralizing and localizing information on interictal F-18 FDG PET in patients with non-lesional TLE can be used as a guide in placing subdural electrodes for invasive EEG monitoring. It has been shown that PET-positive non-lesional TLE patients had excellent post-surgical outcomes after anterior temporal lobectomy, similar to the outcome in patients with mesial temporal sclerosis, even if they did not undergo invasive EEG monitoring (32). Ictal perfusion SPECT study has a high (97%) sensitivity in the localization of the epileptogenic focus in patients with TLE (41). In the ictal perfusion study, TLE patients with a lesion in the mesial temporal lobe show areas of hyperperfusion involving the ipsilateral mesial and lateral temporal regions (27,42). However, the timing of tracer injection is relevant to the area of hyperperfusion. In mesial TLE, an injection of tracer within 15 seconds from the seizure onset would show hyperperfusion of the temporal lobe with accompanying hypoperfusion of the surrounding structures like ipsilateral orbital cortex and ipsilateral frontal lobe (43). A slightly delayed or peri-ictal injection would show hyperperfusion in the ipsilateral lateral temporal cortex, orbital cortex, basal ganglia, motor cortex. Bilateral temporal cortical or generalized increased tracer uptake may also be observed which are features related to the spread of the seizure (27).

## EXTRATEMPORAL LOBE EPILEPSY

**Figure 5:**
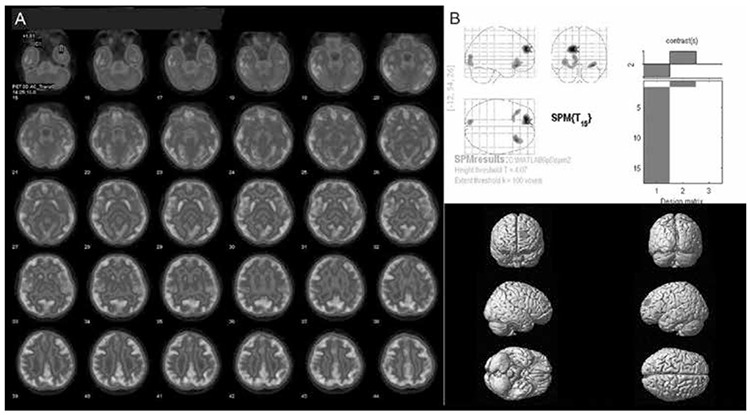
Interictal fluorine-18 fluorodeoxyglucose positron emission tomography study of a patient with epilepsy with cortical dysplasia detected on magnetic resonance imaging in the left frontal and occipital lobes. The axial views (A) show left frontal and occipital cortical hypometabolism visually that were also evident on the quantitative analysis by statistical parametric mapping (B) SPM: Statistical parametric mapping

The localization of the epileptogenic focus in patients with intractable extratemporal lobe epilepsy (ETLE) generally requires more extensive noninvasive and invasive EEG monitoring. The main role of functional imaging with radioisotopes in lesional or non-lesional ETLE is to guide the surgeon in placing the subdural electrodes for invasive EEG monitoring. However, the sensitivity of interictal F-18 FDG PET in detecting the epileptogenic focus in ETLE is not as high as in patients with TLE (30,44). For example, the sensitivity of F-18 FDG PET in frontal lobe epilepsy for localizing the epileptogenic zone was reported to be in the range of 44% to 74% (44,45). The hypometabolic area on F-18 FDG PET generally extends beyond the primary epileptogenic region and patients with chronic partial epilepsy show larger areas of hypometabolism when compared with patients with a new onset partial epilepsy (46). Therefore, F-18 FDG PET is useful for the general localization and lateralization of an epileptogenic focus (Figure 5). The usefulness of ictal SPECT in ETLE is to confirm the epileptogenicity of the structural lesion visible on MRI, and to decide on the area to place subdural electrodes for invasive EEG monitoring in non-lesional patients. However, a limiting factor for ictal SPECT scanning in ETLE is the short duration of seizures. A late tracer injection may show the spread of the seizure rather than the epileptogenic focus. Ictal SPECT is not helpful in secondary generalized seizures because these demonstrate multiple areas of hyperperfusion. In a study which included 117 patients with neocortical epilepsy, the success rates of MRI, PET and ictal SPECT in localization of the epileptogenic focus were reported as 60%, 78%, and 70%, respectively (47).

## NUCLEAR MEDICINE IMAGING IN BRAIN TUMORS

**Figure 6:**
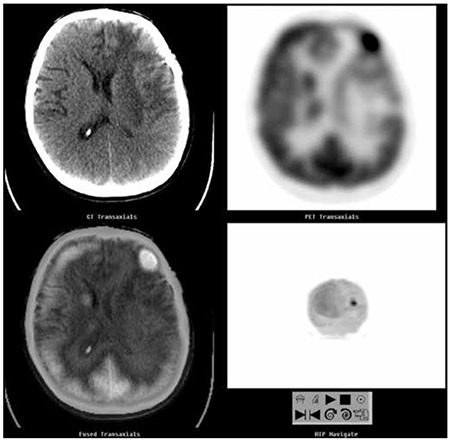
Fluorine-18 fluorodeoxyglucose positron emission tomography/computed tomography (F-18 FDG PET/CT) study of a patient with suspected recurrent astrocytoma of the left frontal lobe. The axial CT, PET and fused PET/CT slices show focal increased F-18 FDG uptake in the recurrent lesion with peripheral cortical hypometabolism due to cerebral edema that is evident on the CT image

Brain tumors are the most common solid tumors in childhood. The management of a child with a brain tumor depends on both the histological features of the tumor and its location within the nervous system. Although surgery is the mainstay of therapy in primary brain tumors, centrally localized tumors can rarely be totally resected and additional chemoradiotherapy is usually required. MRI is generally used to monitor the effects of treatment on the tumor and to detect recurrence. However, MRI is impaired by changes in brain tissue related to surgery and chemoradiotherapy that may cause false positive contrast enhancement. PET and SPECT imagings are suggested for monitoring treatment effects and detecting recurrence (Figure 6). When quantitatively evaluated, the change in F-18 FDG uptake may provide an early and sensitive marker of the effect of chemoradiotherapy (48). F-18 FDG PET can also be useful in differentiating a recurrent tumor from post-treatment changes in children with different brain tumors (49).

F-18 FDG is the most commonly used PET tracer for metabolic studies of brain tumors. It can be used as a diagnostic tool since malignant tumors have a higher F-18 FDG uptake and benign tumors have a lower F-18 FDG uptake than normal brain tissue. Additionally, F-18 FDG PET can be used as a tool to improve the quality of brain tumor biopsies by metabolically showing the most active part of the tumor (50). Although F-18 FDG uptake is positively correlated with tumor grade in childhood brain tumors, an overlap between high-grade and low-grade tumors exists that decreases the specificity of F-18 FDG PET imaging in grading the malignancy (51,52). Semi-quantitative analytic methods that are simpler and not requiring blood sampling are preferred in routine clinical F-18 FDG PET evaluation of brain tumors. Standardized uptake value (SUV) which is defined as the radioactivity in tissue divided by the injected dose multiplied by a patient-specific parameter (body weight, body surface, or lean body mass) is the most commonly used semi-quantitative index in oncologic PET studies. However, SUV was not shown to be useful in grading childhood brain tumors (52), and currently there is no consensus on the optimal quantitative method for brain tumor evaluation with F-18 FDG PET. Determining the ratio of metabolic activity in the tumor to that in the normal structures, such as the contralateral white matter or cortex, the cerebellum or the whole brain, have been used as a means of assessing tumor uptake of F-18 FDG (1,8,50,51,52). Due to the physiological F-18 FDG uptake in normal gray matter, which makes interpretation of F-18 FDG PET studies difficult, other tracers with a greater tumor to background ratio were also investigated as alternative methods in grading malignancy and delineation of tumor extent with SPECT and PET. The radiolabelled amino acids I-123-IMT, C-11-methionine, F-18-fluoroethyltyrosine, F-18-dihydroxyphenylalanine; the phospholipid precursors C-11-choline and F-18-fluorocholine and the thymidine nucleoside analog F-18-fluorothymidine have been shown to be useful in detecting recurrent or residual gliomas when MRI findings were ambiguous as well as in planning and monitoring of therapy (5,9,33,49,50,52,53,54,55,56,57). Other tracers commonly used in imaging pediatric brain tumors are thallium-201 and Tc-99m-methoxyisobutylisonitrile. Both radiotracers showed negligible uptake by the normal brain. Their uptake has been shown to be greater in tumor cells than in normal connective tissue or inflammatory cells, and it is negligible in areas of necrosis (58,59), therefore they can be used in the differentiation of radiation necrosis from recurrent disease, when the MRI is equivocal.

## NUCLEAR MEDICINE IMAGING IN OTHER INDICATIONS

Pediatric brain injury is among the most common causes of death or permanent disability in children. Brain injury can result in various neuropathological changes that affect cerebral blood flow and metabolism; that is why functional neuroimaging may show changes that occur in response to neurologic damage even in regions that appear structurally normal on CT or MRI and provide a more accurate diagnosis of neurologic damage and clinical outcome, especially when there is no clear-cut relationship between the anatomic and clinical findings (60,61,62,63,64,65). Regional cerebral perfusion abnormalities on Tc-99m-HMPAO SPECT were shown to be associated with early anterograde amnesia after mild head trauma in patients with normal CT (60). The brain lesions detected on SPECT scans in the early post-traumatic period may lead to brain atrophy (63). Tc-99m-HMPAO SPECT was found to be a more sensitive method than CT, EEG or MRI for detecting neural lesions and assessing the extent of brain lesion during the chronic stage of pediatric brain injury (64,65,66). The period of metabolic reduction seen on F-18 FDG PET imaging after mild and severe traumatic brain injury generally persists for several weeks (60). However, studies regarding the correlation between neuropsychological deficits and regional metabolic abnormalities have reported inconsistent results (61,67).

**Figure 7:**
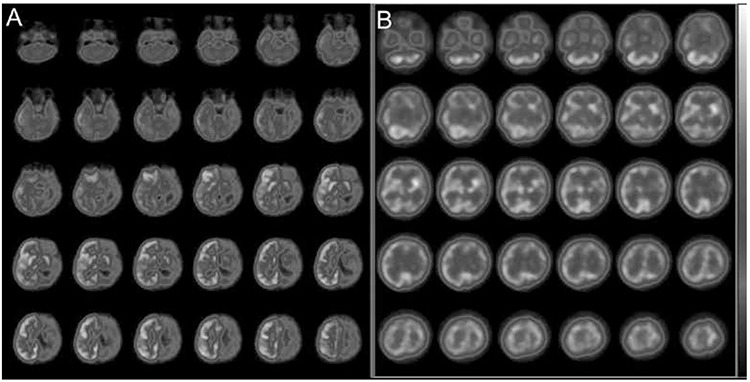
Interictal brain fluorine-18 fluorodeoxyglucose positron emission tomography/computed tomography of a patient with Rasmussen’s syndrome showed extensive hypometabolism in the left cerebral hemisphere involving the ipsilateral basal ganglion and the thalamic nucleus (A). On the ictal Tc-99m hexamethyl propylene amine oxime perfusion single-photon emission computed tomography diffuse increased perfusion in the corresponding regions was observed indicating the distribution of seizure activity (B)

Rasmussen’s encephalitis, which is a childhood disease of unknown etiology and presenting with resistant epilepsy, progressive hemiparesis and cognitive disorders, is among the most commonly investigated inflammatory neurological diseases in children by SPECT and PET. Typically only one hemisphere is affected in Rasmussen’s encephalitis and cortical atrophy develops in the affected hemisphere (Figure 7). Brain SPECT and PET findings may be useful in the diagnosis and surgical treatment of Rasmussen’s encephalitis by determination of affected cortical sites that display decreased perfusion and metabolism (68,69).

In newborn patients with hypoxic-ischemic encephalopathy, brain perfusion SPECT and F-18 FDG PET scan findings have been compared with neuro-developmental outcomes (70,71,72). Studies in term newborns with hypoxic-ischemic encephalopathy have shown that brain perfusion SPECT and F-18 FDG PET findings during the subacute period after perinatal asphyxia correlated with the severity of encephalopathy and short-term clinical outcome (70,71). The most common finding in cerebral blood flow studies was the relative hypoperfusion of the parasagittal regions (72). Additionally, striatal D2 receptor density was shown to be inversely correlated with the severity of injury in infants with hypoxic-ischemic encephalopathy, and higher D2 receptor density was found in infants who recovered without neurologic deficits (73).

## CONCLUSION

Nuclear medicine methods can contribute to the management of pediatric patients with neurological diseases. Previous studies have demonstrated that nuclear medicine imaging provided complementary information in pre-surgical localization of the epileptogenic focus in medically refractory epilepsy patients as well as in diagnosis and surveillance of patients with brain tumors. Other indications of nuclear medicine in pediatric neurology are the evaluation of patients with head trauma, inflammatory-infectious central nervous system diseases, and hypoxic-ischemic encephalopathy. Although brain perfusion and metabolism are the most commonly studied functions, several other more specific tracers exist for assessment of various neuronal functions.

## References

[1] Meyer PT, Schreckenberger M, Spetzger U, Meyer GF, Sabri O, Setani KS, Zeggel T, Buell U (2001). Comparison of visual and ROI-based brain tumour grading using 18F-FDG PET: ROC analyses. Eur J Nucl Med.

[2] Kapucu OL, Nobili F, Varrone A, Booij J, Vander Borght T, Nagren K, Darcourt J, Tatsch K, Van Laere KJ (2009). EANM procedure guideline for brain perfusion SPECT using 99mTc-labelled radiopharmaceuticals, version 2. Eur J Nucl Med Mol Imaging.

[3] Saha GB, MacIntyre WJ, Go RT (1994). Radiopharmaceuticals for brain imaging. Semin Nucl Med.

[4] Grünwald F, Menzel C, Pavics L, Bauer J, Hufnagel A, Reichmann K, Sakowski R, Elger CE, Biersack HJ (1994). Ictal and interictal brain SPECT imaging in epilepsy using technetium-99m-ECD. J Nucl Med.

[5] Tripathi M, Sharma R, D’Souza M, Jaimini A, Panwar P, Varshney R, Datta A, Kumar N, Garg G, Singh D, Grover RK, Mishra AK, Mondal A (2009). Comparative evaluation of F-18 FDOPA, F-18 FDG, and F-18 FLT-PET/CT for metabolic imaging of low grade gliomas. Clin Nucl Med.

[6] Van Laere K, Varrone A, Booij J, Vander Borght T, Nobili F, Kapucu OL, Walker Z, Nagren K, Tatsch K, Darcourt J (2010). EANM procedure guidelines for brain neurotransmission SPECT/PET using dopamine D2 receptor ligands, version 2. Eur J Nucl Med Mol Imaging.

[7] Darcourt J, Booij J, Tatsch K, Varrone A, Vander Borght T, Kapucu OL, Nagren K, Nobili F, Walker Z, Van Laere K (2010). EANM procedure guidelines for brain neurotransmission SPECT using (123)I-labelled dopamine transporter ligands, version 2. Eur J Nucl Med Mol Imaging.

[8] Varrone A, Asenbaum S, Vander Borght T, Booij J, Nobili F, Nagren K, Darcourt J, Kapucu OL, Tatsch K, Bartenstein P, Van Laere K (2009). EANM procedure guidelines for PET brain imaging using [18F]FDG, version 2. Eur J Nucl Med Mol Imaging.

[9] Zimmer L, Luxen A (2012). PET radiotracers for molecular imaging in the brain: past, present and future. Neuroimage.

[10] Varghese GI, Purcaro MJ, Motelow JE, Enev M, McNally KA, Levin AR, Hirsch LJ, Tikofsky R, Paige AL, Zubal IG, Spencer SS, Blumenfeld H (2009). Clinical use of ictal SPECT in secondarily generalized tonic-clonic seizures. Brain.

[11] Drzezga A, Arnold S, Minoshima S, Noachtar S, Szecsi J, Winkler P, Römer W, Tatsch K, Weber W, Bartenstein P (1999). 18F-FDG PET studies in patients with extratemporal and temporal epilepsy: evaluation of an observer-independent analysis. J Nucl Med.

[12] Kumar A, Juhasz C, Asano E, Sood S, Muzik O, Chugani HT (2010). Objective detection of epileptic foci by 18F-FDG PET in children undergoing epilepsy surgery. J Nucl Med.

[13] Lin TW, Aburto MA, Dahlbom M, Huang LL, Marvi MM, Tang M, Czernin J, Phelps ME, Silverman DH (2007). Predicting seizure-free status for temporal lobe epilepsy patients undergoing surgery: prognostic value of quantifying maximal metabolic asymmetry extending over a specified proportion of the temporal lobe. J Nucl Med.

[14] Lee JY, Joo EY, Park HS, Song P, Young Byun S, Seo DW, Hong SB (2011). Repeated ictal SPECT in partial epilepsy patients: SISCOM analysis. Epilepsia.

[15] Kazemi NJ, Worrell GA, Stead SM, Brinkmann BH, Mullan BP, O’Brien TJ, So EL (2010). Ictal SPECT statistical parametric mapping in temporal lobe epilepsy surgery. Neurology.

[16] Van Paesschen W (2004). Ictal SPECT. Epilepsia.

[17] O’Brien TJ, So EL, Mullan BP, Hauser MF, Brinkmann BH, Bohnen NI, Hanson D, Cascino GD, Jack CR, Sharbrough FW (1998). Subtraction ictal SPECT co-registered to MRI improves clinical usefulness of SPECT in localizing the surgical seizure focus. Neurology.

[18] O’Brien TJ, So EL, Cascino GD, Hauser MF, Marsh WR, Meyer FB, Sharbrough FW, Mullan BP (2004). Subtraction SPECT coregistered to MRI in focal malformations of cortical development: localization of the epileptogenic zone in epilepsy surgery candidates. Epilepsia.

[19] Tan KM, Britton JW, Buchhalter JR, Worrell GA, Lagerlund TD, Shin C, Cascino GD, Meyer FB, So EL (2008). Influence of subtraction ictal SPECT on surgical management in focal epilepsy of indeterminate localization: a prospective study. Epilepsy Res.

[20] Seo JH, Holland K, Rose D, Rozhkov L, Fujiwara H, Byars A, Arthur T, DeGrauw T, Leach JL, Gelfand MJ, Miles L, Mangano FT, Horn P, Lee KH (2011). Multimodality imaging in the surgical treatment of children with nonlesional epilepsy. Neurology.

[21] Lee JJ, Kang WJ, Lee DS, Lee JS, Hwang H, Kim KJ, Hwang YS, Chung JK, Lee MC (2005). Diagnostic performance of 18F-FDG PET and ictal 99mTc-HMPAO SPET in pediatric temporal lobe epilepsy: quantitative analysis by statistical parametric mapping, statistical probabilistic anatomical map, and subtraction ictal SPET. Seizure.

[22] Tae WS, Joo EY, Kim JH, Han SJ, Suh YL, Kim BT, Hong SC, Hong SB (2005). Cerebral perfusion changes in mesial temporal lobe epilepsy: SPM analysis of ictal and interictal SPECT. Neuroimage.

[23] Bruggemann JM, Som SS, Lawson JA, Haindl W, Cunningham AM, Bye AM (2004). Application of statistical parametric mapping to SPET in the assessment of intractable childhood epilepsy. Eur J Nucl Med Mol Imaging.

[24] Koç E, Serdaroğlu A, Kapucu O, Atalay Y, Gücüyener K, Atasever T (1997). Ictal and interictal SPECT in a newborn infant with intractable seizure. Acta Paediatr.

[25] Chugani HT (1998). A critical period of brain development: studies of cerebral glucose utilization with PET. Prev Med.

[26] Sood S, Chugani HT (2006). Functional neuroimaging in the preoperative evaluation of children with drug-resistant epilepsy. Childs Nerv Syst.

[27] Goffin K, Dedeurwaerdere S, Van Laere K, Van Paesschen W (2008). Neuronuclear assessment of patients with epilepsy. Semin Nucl Med.

[28] Kapucu LO, Serdaroğlu A, Okuyaz C, Köse G, Gücüyener K (2003). Brain single photon emission computed tomographic evaluation of patients with childhood absence epilepsy. J Child Neurol.

[29] Kapucu LO, Gücüyener K, Vural G, Köse G, Tokçaer AB, Turgut B, Unlü M (1996). Brain SPECT evaluation of patients with pure photosensitive epilepsy. J Nucl Med.

[30] Casse R, Rowe CC, Newton M, Berlangieri SU, Scott AM (2002). Positron emission tomography and epilepsy. Mol Imaging Biol.

[31] O’Brien TJ, Miles K, Ware R, Cook MJ, Binns DS, Hicks RJ (2008). The cost-effective use of 18F-FDG PET in the presurgical evaluation of medically refractory focal epilepsy. J Nucl Med.

[32] LoPinto-Khoury C, Sperling MR, Skidmore C, Nei M, Evans J, Sharan A, Mintzer S (2012). Surgical outcome in PET-positive, MRI-negative patients with temporal lobe epilepsy. Epilepsia.

[33] Patil S, Biassoni L, Borgwardt L (2007). Nuclear medicine in pediatric neurology and neurosurgery: epilepsy and brain tumors. Semin Nucl Med.

[34] Capraz IY, Kurt G, Akdemir Ö, Hirfanoglu T, Oner Y, Sengezer T, Kapucu LO, Serdaroglu A, Bilir E (2015). Surgical outcome in patients with MRI-negative, PET-positive temporal lobe epilepsy. Seizure.

[35] Wu JY, Salamon N, Kirsch HE, Mantle MM, Nagarajan SS, Kurelowech L, Aung MH, Sankar R, Shields WD, Mathern GW (2010). Noninvasive testing, early surgery, and seizure freedom in tuberous sclerosis complex. Neurology.

[36] Rastogi S, Lee C, Salamon N (2008). Neuroimaging in pediatric epilepsy: a multimodality approach. Radiographics.

[37] Koepp MJ, Woermann FG (2005). Imaging structure and function in refractory focal epilepsy. Lancet Neurol.

[38] Akimura T, Yeh HS, Mantil JC, Privitera MD, Gartner M, Tomsick TA (1999). Cerebral metabolism of the remote area after epilepsy surgery. Neurol Med Chir (Tokyo).

[39] Blum DE, Ehsan T, Dungan D, Karis JP, Fisher RS (1998). Bilateral temporal hypometabolism in epilepsy. Epilepsia.

[40] Delbeke D, Lawrence SK, Abou-Khalil BW, Blumenkopf B, Kessler RM (1996). Postsurgical outcome of patients with uncontrolled complex partial seizures and temporal lobe hypometabolism on 18FDG-positron emission tomography. Invest Radiol.

[41] Devous MD, Thisted RA, Morgan GF, Leroy RF, Rowe CC (1998). SPECT brain imaging in epilepsy: a meta-analysis. J Nucl Med.

[42] Ho SS, Berkovic SF, McKay WJ, Kalnins RM, Bladin PF (1996). Temporal lobe epilepsy subtypes: differential patterns of cerebral perfusion on ictal SPECT. Epilepsia.

[43] Newton MR, Berkovic SF, Austin MC, Rowe CC, McKay WJ, Bladin PF (1992). Postictal switch in blood flow distribution and temporal lobe seizures. J Neurol Neurosurg Psychiatry.

[44] Lee SK, Lee SY, Kim KK, Hong KS, Lee DS, Chung CK (2005). Surgical outcome and prognostic factors of cryptogenic neocortical epilepsy. Ann Neurol.

[45] Kim YK, Lee DS, Lee SK, Chung CK, Chung JK, Lee MC (2002). (18)F-FDG PET in localization of frontal lobe epilepsy: comparison of visual and SPM analysis. J Nucl Med.

[46] Matheja P, Kuwert T, Ludemann P, Weckesser M, Kellinghaus C, Schuierer G, Diehl B, Ringelstein EB, Schober O (2001). Temporal hypometabolism at the onset of cryptogenic temporal lobe epilepsy. Eur J Nucl Med.

[47] Hwang SI, Kim JH, Park SW, Han MH, Yu IK, Lee SH, Lee DS, Lee SK, Chung CK, Chang KH (2001). Comparative analysis of MR imaging, positron emission tomography, and ictal single-photon emission CT in patients with neocortical epilepsy. AJNR Am J Neuroradiol.

[48] Hölzer T, Herholz K, Jeske J, Heiss WD (1993). FDG-PET as a prognostic indicator in radiochemotherapy of glioblastoma. J Comput Assist Tomogr.

[49] Plowman PN, Saunders CA, Maisey M (1997). On the usefulness of brain PET scanning to the paediatric neuro-oncologist. Br J Neurosurg.

[50] Wong TZ, Coleman RE (2002). Positron emission tomography imaging of brain tumors. Neuroimaging Clin N Am.

[51] Borgwardt L, Hojgaard L, Carstensen H, Laursen H, Nowak M, Thomsen C, Schmiegelow K (2005). Increased fluorine-18 2-fluoro-2-deoxy-D-glucose (FDG) uptake in childhood CNS tumors is correlated with malignancy grade: a study with FDG positron emission tomography/magnetic resonance imaging coregistration and image fusion. J Clin Oncol.

[52] Utriainen M, Metsahonkala L, Salmi TT, Utriainen T, Kalimo H, Pihko H, Makipernaa A, Harila-Saari A, Jyrkkio S, Laine J, Nagren K, Minn H (2002). Metabolic characterization of childhood brain tumors: comparison of 18F-fluorodeoxyglucose and 11C-methionine positron emission tomography. Cancer.

[53] Galldiks N, Kracht LW, Berthold F, Miletic H, Klein JC, Herholz K, Jacobs AH, Heiss WD (2010). [11C]-L-methionine positron emission tomography in the management of children and young adults with brain tumors. J Neurooncol.

[54] Peet AC, Leach MO, Pinkerton CR, Price P, Williams SR, Grundy RG (2005). The development of functional imaging in the diagnosis, management and understanding of childhood brain tumours. Pediatr Blood Cancer.

[55] Kasper BS, Struffert T, Kasper EM, Fritscher T, Pauli E, Weigel D, Kerling F, Hammen T, Graf W, Kuwert T, Prante O, Lorber B, Buchfelder M, Doerfler A, Schwab S, Stefan H, Linke R (2011). 18Fluoroethyl-L-tyrosine-PET in long-term epilepsy associated glioneuronal tumors. Epilepsia.

[56] Pöpperl G, Götz C, Rachinger W, Gildehaus FJ, Tonn JC, Tatsch K (2004). Value of O-(2-[18F]fluoroethyl)- L-tyrosine PET for the diagnosis of recurrent glioma. Eur J Nucl Med Mol Imaging.

[57] Pirotte B, Goldman S, Massager N, David P, Wikler D, Lipszyc M, Salmon I, Brotchi J, Levivier M (2004). Combined use of 18F-fluorodeoxyglucose and 11C-methionine in 45 positron emission tomography-guided stereotactic brain biopsies. J Neurosurg.

[58] Maria BL, Drane WE, Mastin ST, Jimenez LA (1998). Comparative value of thallium and glucose SPECT imaging in childhood brain tumors. Pediatr Neurol.

[59] Kirton A, Kloiber R, Rigel J, Wolff J (2002). Evaluation of pediatric CNS malignancies with (99m)Tc-methoxyisobutylisonitrile SPECT. J Nucl Med.

[60] Lorberboym M, Lampl Y, Gerzon I, Sadeh M (2002). Brain SPECT evaluation of amnestic ED patients after mild head trauma. Am J Emerg Med.

[61] Bergsneider M, Hovda DA, McArthur DL, Etchepare M, Huang SC, Sehati N, Satz P, Phelps ME, Becker DP (2001). Metabolic recovery following human traumatic brain injury based on FDG-PET: time course and relationship to neurological disability. J Head Trauma Rehabil.

[62] Alavi A (1989). Functional and anatomic studies of head injury. J Neuropsychiatry Clin Neurosci.

[63] Hofman PA, Stapert SZ, Kroonenburgh MJ, Jolles J, Kruijk J, Wilmink JT (2001). MR imaging, single-photon emission CT, and neurocognitive performance after mild traumatic brain injury. AJNR Am J Neuroradiol.

[64] Goshen E, Zwas ST, Shahar E, Tadmor R (1996). The role of 99Tcm-HMPAO brain SPET in paediatric traumatic brain injury. Nucl Med Commun.

[65] Emanuelson IM, von Wendt L, Bjure J, Wiklund LM, Uvebrant P (1997). Computed tomography and single-photon emission computed tomography as diagnostic tools in acquired brain injury among children and adolescents. Dev Med Child Neurol.

[66] Gowda NK, Agrawal D, Bal C, Chandrashekar N, Tripati M, Bandopadhyaya GP, Malhotra A, Mahapatra AK (2006). Technetium Tc-99m ethyl cysteinate dimer brain single-photon emission CT in mild traumatic brain injury: a prospective study. AJNR Am J Neuroradiol.

[67] Worley G, Hoffman JM, Paine SS, Kalman SL, Claerhout SJ, Boyko OB, Kandt RS, Santos CC, Hanson MW, Oakes WJ (1995). 18-Fluorodeoxyglucose positron emission tomography in children and adolescents with traumatic brain injury. Dev Med Child Neurol.

[68] Geller E, Faerber EN, Legido A, Melvin JJ, Hunter JV, Wang Z, Chadarevian JP (1998). Rasmussen encephalitis: complementary role of multitechnique neuroimaging. AJNR Am J Neuroradiol.

[69] Ishibashi H, Simos PG, Wheless JW, Baumgartner JE, Kim HL, Davis RN, Zhang W, Papanicolaou AC (2002). Multimodality functional imaging evaluation in a patient with Rasmussen’s encephalitis. Brain Dev.

[70] Shi Y, Zhao JN, Liu L, Hu ZX, Tang SF, Chen L, Jin RB (2012). Changes of positron emission tomography in newborn infants at different gestational ages, and neonatal hypoxic-ischemic encephalopathy. Pediatr Neurol.

[71] Thorngren-Jerneck K, Ohlsson T, Sandell A, Erlandsson K, Strand SE, Ryding E, Svenningsen NW Cerebral glucose metabolism measured by positron emission tomography in term newborn infants with hypoxic ischemic encephalopathy. Pediatr Res 2001.

[72] Shah S, Fernandez AR, Chirla D (2001). Role of brain SPECT in neonates with hypoxic ischemic encephalopathy and its correlation with neurodevelopmental outcome. Indian Pediatr.

[73] Kapucu LO, Koc E, Gücüyener K, Zenciroğlu A, Atalay Y, Unlü M, Royen E (1998). D2 receptor imaging with iodine-123-iodobenzamide brain SPECT in infants with hypoxic-ischemic brain injury. J Nucl Med.

